# Evaluation of oxidative stress and antioxidant defense biomarkers in healthy and colic horses: correlation with type of colic and outcome

**DOI:** 10.1093/jvimsj/aalag017

**Published:** 2026-02-14

**Authors:** Francesca Bindi, Lucia de Marchi, Ane Elias-Cortajarena, Giulia Sala, Valentina Vitale, Alessandro Spadari, Riccardo Rinnovati, Francesca Bonelli, Micaela Sgorbini

**Affiliations:** Department of Veterinary Science, University of Pisa, San Piero a Grado, Pisa 56122, Italy; Department of Veterinary Science, University of Pisa, San Piero a Grado, Pisa 56122, Italy; Hospital Clínico Veterinario, Universidad CEU-Cardenal Herrera, CEU Universities, Alfara del Patriarca, Valencia 46115, Spain; Department of Veterinary Science, University of Pisa, San Piero a Grado, Pisa 56122, Italy; Hospital Clínico Veterinario, Universidad CEU-Cardenal Herrera, CEU Universities, Alfara del Patriarca, Valencia 46115, Spain; Department of Veterinary Medical Sciences, Alma Mater Studiorum—University of Bologna, Ozzano dell'Emilia, Bologna 40064, Italy; Department of Veterinary Medical Sciences, Alma Mater Studiorum—University of Bologna, Ozzano dell'Emilia, Bologna 40064, Italy; Department of Veterinary Science, University of Pisa, San Piero a Grado, Pisa 56122, Italy; Department of Veterinary Science, University of Pisa, San Piero a Grado, Pisa 56122, Italy

**Keywords:** antioxidant defense, oxidative stress, prognostic biomarkers, SIRS

## Abstract

**Background:**

Colic is a major cause of morbidity and mortality in horses, with oxidative stress implicated in its pathophysiology.

**Hypothesis/Objectives:**

Evaluate biomarkers (BIOs) of oxidative stress and antioxidant defense in healthy horses and those with non-strangulating colic (NSC) and strangulating colic (SC) and assess correlations with survival.

**Animals:**

Seventy-one adult horses: 10 healthy and 61 colic-affected (42 NSC, 19 SC) admitted to 3 veterinary teaching hospitals.

**Methods:**

Prospective, multicenter cohort study. Blood samples were collected at admission (T0) and up to 96 h post-admission. Biomarkers measured included arylesterase (AREase), paraoxonase (POase), lipid peroxidation (LPO), superoxide dismutase (SOD), butyrylcholinesterase, total antioxidant capacity (TAC), glutathione S-transferase (GST), and glutathione peroxidase (GPx). Data were analyzed using nonparametric statistics and generalized linear mixed models.

**Results:**

Compared with healthy horses, colic-affected horses had higher AREase (*P* = .01), GST (*P* = .001), and GPx (*P* = .001), and lower POase (*P* < .001) and TAC (*P* = .02). Survival was associated with higher AREase (coefficient [coef.] 106.65 kU/L; 95% confidence interval [CI], 24.70-188.60; *P* = .01), lower SOD (coef. −0.38 U/mL; 95%CI, −0.76 to −0.06; *P* = .03), and lower TAC (coef. −3.37 μmol/mL; 95%CI, −5.49 to −1.25; *P* = .01). Colic type also influenced results, with NSC (vs SC) associated with lower LPO (coef. −1.24 malondialdehyde [MDA]/μL; 95%CI, −2.81 to −0.32; *P* = .01), higher SOD (coef. 0.42; 95%CI, 0.03-0.81; *P* = .04), and higher TAC (coef. 1.21; 95%CI, 0.10-2.98; *P* = .04).

**Conclusions and clinical importance:**

Results emphasize the association between oxidative stress BIOs and colic in horses, suggesting that specific BIOs, particularly AREase, may have prognostic utility.

## Introduction

Colic syndrome is among the most frequently encountered emergencies in equine veterinary practice and remains a leading cause of mortality in horses.^1^ This condition is characterized by intense abdominal pain and associated with substantial hemodynamic alterations arising from tissue injuries caused by inflammatory or infectious processes.^2^ One of the primary causes of mortality in horses with colic is endotoxemia, often resulting from the absorption of bacterial lipopolysaccharides after ischemic gut injury and rapid breakdown of the intestinal barrier.^3^^,^^4^ Oxidative stress, defined as an imbalance between oxidants and antioxidants, leads to oxidative damage characterized by cellular macromolecule modification, apoptosis, necrosis, and structural tissue damage.^5^

Despite increasing interest in the role of oxidative stress in colic in horses, studies investigating associated biomarkers (BIOs) remain limited and often focus on a narrow subset of markers or specific clinical scenarios. The role of paraoxonase 1 (PON1), particularly its paraoxonase (POase) activity, has been increasingly explored in horses with inflammation,^6–8^ but its utility in colic, especially in relation to outcome, has not been fully established. In contrast, the arylesterase (AREase) activity of PON1 has not yet been investigated in horses. Other markers such as butyrylcholinesterase (BChE), which may modulate inflammation via the cholinergic anti-inflammatory pathway,^9^ are still poorly characterized in this species. Lipid peroxidation (LPO) activity has been evaluated in horses with colic, with 1 study reporting that their activity may vary depending on the type of colic.^10^ Superoxide dismutase (SOD) activity and total antioxidant capacity (TAC) activity recently have been investigated in gastrointestinal disorders such as peritonitis,^11^ enterocolitis,^12^ colitis,^13^ and equine gastric ulcer syndrome (EGUS).^14^ In addition, glutathione S-transferase (GST) has been studied in human patients with intestinal ischemia,^15–17^ whereas glutathione peroxidase (GPx) has not been widely applied to gastrointestinal disease in horses. Nevertheless, both GST and GPx have been used as indicators of oxidative stress in horses subjected to prolonged transportation and strenuous physical exercise.^18–20^

The relationship between these BIOs and the type of colic (non-strangulating colic [NSC] vs strangulating colic [SC]) as well as their biological relevance, remains unclear. A broader, integrated evaluation of oxidative stress and antioxidant BIOs is needed to provide a comprehensive overview of the oxidative/antioxidant status in horses with colic.

Our primary aim was to evaluate and describe differences in BIOs involved in the antioxidant defense system and oxidative stress response between healthy horses and those affected by colic, including comparisons between NSC and SC. Our secondary aim was to assess how BIOs activity or concentration varied with clinical outcome, defined as survival or non-survival, to gain insight into the oxidative and antioxidant responses associated with disease progression.

## Materials and methods

Our in vivo, prospective, multicenter study was approved by the Ethical Committee of the University of Pisa (Prot. N. 03/2023) and written owner consent was obtained for plasma collection for all the horses in this study. The study was performed over a 2-year period.

### Study population and sampling procedure

Horses referred for evaluation of colic to 3 different veterinary teaching hospitals (VTHs) providing secondary health care were enrolled. The VTHs followed the same protocols for diagnosis of colic and treatment.

Horses with a diagnosis of either NSC or SC were enrolled because they represent the principal caseload of the VTHs involved in the study. The diagnosis of gastrointestinal colic was based on signalment, clinical history, and physical examination findings. The latter included the assessment of pain status and vital signs, nasogastric intubation, abdominal auscultation, and abdominal palpation per rectum. Abdominal ultrasonography and abdominocentesis also were performed when indicated. In horses undergoing surgical treatment, intraoperative findings at laparotomy were used to confirm the diagnosis. A total and differential white blood cell count was performed to assess the presence and magnitude of an inflammatory response process contributing to the pathophysiology of the colic syndrome. Venous blood gases and lactate concentration were assessed to provide information about acid–base status.^21^

A cohort of mares owned by 1 of the 3 VTHs and used for reproduction purposes in a commercial embryo transfer activity was used as the healthy group to provide baseline reference values for oxidative stress and antioxidant BIOs. To be included in the healthy group, mares had to be clinically healthy based on a normal physical examination, and with CBC and blood lactate concentration within reference ranges. Horses from the healthy group were regularly vaccinated and dewormed with no treatment at least 15 days before the study was performed.

In horses with colic signs, the following data were recorded at T0 to evaluate systemic inflammatory response syndrome (SIRS) status^22^: presence of abnormal leukocyte count or distribution as leukopenia, leukocytosis (<5 × 10^3^/μL or > 12.5 × 10^3^/μL) or > 10% band neutrophils, hyperthermia or hypothermia (<37°C or > 38.5°C), tachycardia (>52 beats per min), and tachypnea (>20 breaths per min). Horses with 0 or 1 abnormal criterion were considered SIRS-negative, whereas those with ≥ 2 abnormal criteria were considered SIRS-positive. Moreover, blood lactate concentration (normal ≤ 2.06 mmol/L vs > 2.06 mmol/L) and color of the mucous membranes (normal mucous membranes: pink and moist vs abnormal mucous membranes: injected, purple, muddy, toxic, red, or white) were evaluated to calculate the 0-6 point-scale SIRS score.^22^

Finally, colic horses were retrospectively divided into a survivor group and a non-survivor group. The non-survivor horses spontaneously died or were euthanized because of worsening clinical signs and not because of financial limitations or other owner’s criteria.

Healthy horses underwent blood sampling once, whereas in horses with signs of colic, blood was collected at admission (T0), then at 24-h intervals until discharge or death for a maximum of 96 h (T1, T2, T3, and T4). Blood samples were collected from the jugular vein using a sterile syringe and 18G needle. A 1 mL aliquot was collected in a potassium EDTA test tube and analyzed by a cell counter (ProCyte Dx, IDEXX Laboratories Italia s.r.l., Milan, Italy) within 5 min after collection. A second 2.5 mL aliquot was collected in lithium-heparinized tubes and centrifuged at 3000 × *g* for 10 min within 30 min of collection. The harvested plasma was placed in sterile tubes, and frozen at −80°C. Biomarkers were measured in multiple batches, as described below. Only at T0, a single drop of whole blood was immediately used to measure blood lactate concentrations using a blood gas analyzer (Stat Profile Primevet, Nova Biochemicals, Waltham, USA).

### Biomarkers evaluation

All laboratory assays were performed by an experienced biologist (L.D.M.) using a spectrophotometric method and a Synergy HT Absorbance Microplate Reader (BioTek Co., USA). All biological samples were stored at −80°C immediately after collection and remained frozen until the time of analysis. All samples were analyzed in triplicate.

The analysis of POase activity was performed following a previously described method^23^ with some modifications. Activity was assessed by spectrophotometric quantification of p-nitrophenol. Briefly, glycine buffer (50.0 mM) containing 1 mM of CaCl_2_ (pH 10.5) and 1.0 mM of paraoxon substrate was added to the undiluted lithium-heparinized plasma sample. This step was followed by incubation at 37°C for 10 min. Absorbance was measured at 412 nm, and activity was expressed as μmol of p-nitrophenol/mL/min.

The AREase activity was assessed following the same modified methodology used for POase.^23^ The method is based on the production of acetic acid through the hydrolysis of phenyl acetate. First, a calibration curve (with concentrations ranging from 5.50 to 26.21 mM) was created from a 30 mM stock solution of acetic acid and phenol prepared in a 4-(2-hydroxyethyl)-1-piperazineethanesulfonic acid (HEPES) buffer (2.0 mM, pH 8.0) containing calcium chloride and bovine serum albumin (BSA, 0.005%). Lithium-heparinized samples were diluted (v/v 1:5) with a saline solution, and freshly prepared HEPES buffer, phenol red (106 mM), and phenyl acetate (5.0 mM) were added. The samples then were incubated at 37°C for 10 min. The absorbance was measured at 405 nm and the obtained activity was expressed in kU/L.

The LPO activity was determined by measuring the amount of malondialdehyde (MDA) in the samples using a modification of a previously described method.^24^ Briefly, lithium-heparinized plasma samples were diluted with distilled water (v/v 1:5) and treated with thiobarbituric acid (TBA)–trichloroacetic acid (TCA)–HCl reagent (0.37% TBA, 15% TCA, and 0.25 N HCl), and then covered with film and brought to a boil for 15 min at 100°C in a water bath. The samples then were cooled in a refrigerator at 4°C for another 15 min and subsequently centrifuged at 3000 × *g* for 3 min at room temperature. The absorbance of the supernatant was measured at 532 nm, and the activity was expressed as μmol of MDA/μL.

The SOD activity was assessed using a modification of a previously described method.^25^ In summary, lithium-heparinized plasma samples were diluted with distilled water (v/v 1:5) and treated with a reaction mixture (xanthine, nitro blue tetrazolium, and phosphate buffer). Subsequently, xanthine oxidase was added to the mixture, and the reaction was stopped by adding copper dichloride (6 mM). The absorbance was measured at 560 nm, and the results were reported as U/mL.

The BTChE activity was investigated according to a modification of a previously described method.^26^ A reaction mixture containing 5,5′-dithiobis(2-nitrobenzoic acid) and 0.1 M phosphate buffer (pH 8.0) was added to the undiluted lithium-heparinized plasma sample. After a 20-min incubation at 25°C, a solution of butyrylthiocholine iodide was added as the substrate. The absorbance was measured at 412 nm for 3 min, and the activity was expressed as μmol/mL/min.

The TAC was assessed according to a modification of a previously described method.^27^ This method measures the capacity of antioxidants to reduce ferric iron. It is based on the reduction of the ferric iron complex with 2,3,5-triphenyl-1,3,4-triaza-2-azoniacyclopenta-1,4-diene chloride (TPTZ) to the ferrous form at low pH. The lithium-heparinized plasma sample was treated with a reaction solution composed of acetate buffer, TPTZ, and ferric chloride. The absorbance was measured at 590 nm, and the activity was expressed as μmol/mL.

The GST activity was measured following a modification of a previously described method,^28^ using 1-chloro-2,4-dinitrobenzene (CDNB) as the substrate. Lithium-heparinized plasma samples were treated with a reaction solution containing 100 mM Tris buffer (pH 7.4), CDNB (1 mM), and reduced glutathione (GSH, 1 mM). The absorbance was measured at 340 nm, and the activity was expressed as U/mg protein.

The GPx activity was analyzed using a previously described method^29^ with some modifications according to another study.^30^ The lithium-heparinized plasma sample was treated with a reaction solution containing 50 mM potassium phosphate buffer (pH 8.3), 1 mM EDTA, 1 mM sodium azide, 0.2 mM nicotinamide adenine dinucleotide phosphate, and 1 U/mL glutathione reductase. The reaction was initiated by adding 1.5 mM cumene hydroperoxide. The absorbance was measured at 340 nm, and the activity was expressed as U/mg protein.

Protein concentration was determined according to a previously described method^31^ using BSA as the standard.

### Statistical analysis

Statistical analysis was carried out using IBM SPSS version 29.0 (IBM Corporation, Armonk, NY, USA). Descriptive statistics were calculated separately for each group (healthy, NSC, SC, SIRS-positive, SIRS-negative, and outcome) and for different sampling times. The Shapiro–Wilk test was applied to assess the distribution of continuous variables, determining that BIOs were not normally distributed, and thus data were presented as the median with the 25th and 75th percentiles.

Comparisons of BIOs between healthy and colic-affected horses, as well as between survivors and non-survivors, were performed using the Mann–Whitney *U* test. Differences in BIOs among the groups (healthy, NSC, and SC) were analyzed using the Kruskal–Wallis test, followed by post hoc pairwise comparisons with Bonferroni correction. The Kruskal–Wallis test also was employed to assess differences in BIOs concentrations across SIRS score categories.

Within the colic group, a generalized linear mixed model (GLMM) was built for each BIO (dependent variable). Fixed effects included colic type (NSC vs SC), SIRS status (positive vs negative), survival outcome (survival vs non-survival), and sampling time. The hospital of origin was included as a random intercept to account for clustering because of the multicenter design, and horse ID was modeled as a repeated effect to account for within-subject correlation over time. Models were fitted assuming a normal distribution with identity link for the response. Model fit and assumptions were evaluated using Akaike’s information criterion (AIC). Although survival outcome was included as an independent variable, it was done for descriptive purposes only to explore whether BIO activities differed between survivors and non-survivors. The model was not intended to infer causality or to predict survival, but rather to provide a descriptive framework for comparing the biochemical profiles across clinical categories. Model fit was evaluated using the AIC, and significance was set at *P* < .05. Given that the BIOs evaluated represent biologically distinct components of oxidative and antioxidant responses, each statistical comparison addressed an independent biological question. Because the study was exploratory in nature and aimed at describing BIOs behavior across different clinical conditions, *P*-values were not adjusted for multiple testing. As a consequence, statistical findings should be interpreted as hypothesis-generating rather than confirmatory.

A post hoc power analysis was performed using G*Power software (version 3.1, Heinrich Heine University, Düsseldorf, Germany), selecting the *t*-tests family and the “two independent groups, nonparametric: Wilcoxon–Mann–Whitney test” option, based on the effect sizes derived from the Mann–Whitney *U* statistics. Large effect sizes were identified for several BIOs (POase *f* = 0.603, GST *f* = 0.585, GPx *f* = 0.540, and lactate *f* = 0.718), yielding post hoc power estimates > 0.80. This result indicates that the available sample size was sufficient to detect large differences between healthy and colic horses. In contrast, BIOs with small effect sizes (including SOD, LPO, TAC, and BChE) were underpowered, further supporting the exploratory and hypothesis-generating nature of these findings.

## Results

Seventy-one adult horses were enrolled. Table 1 summarizes the signalment and clinical characteristics of the enrolled horses, including the subdivision of the colic group into NSC and SC based on final diagnoses.

**Table 1:** Sex distribution, median age (range), and breed composition of healthy horses (*n* = 10) and horses with colic (*n* = 61). Horses with colic are further classified by final diagnosis into non-strangulating colic (NSC) and strangulating colic (SC) groups.

Group	Sex	Age (years old)	Breed	Colic type
**Healthy (*n* = 10)**	10 (100%) mares	12 (7-15)	7 (70%) Standardbred 3 (30%) Thoroughbred	—
**Colic (*n* = 61)**	26 (42.6%) mares 18 (29.5%) geldings 17 (27.9%) stallions	13 (0.5-30)	46 (75.4%) Warmblood 6 (9.8%) Standardbred 4 (6.6%) Thoroughbred 3 (4.9%) ponies 2 (3.3%) draft horses	42 (68.9%) NSC 19 (31.1%) SC

Among horses with NSC, 27/42 (64.3%) received medical treatment, whereas 15/42 (35.7%) underwent surgery. In the SC group, 16/19 (84.2%) were treated surgically and 3/19 (15.8%) received medical treatment. All surgical procedures were performed within the first 24 h after hospital admission. Of the horses affected by NSC, 39/42 (92.9%) were survivors and 3/42 (7.1%) non-survivors. Among those suffering from SC, 12/19 (63.2%) were survivors and 7/19 (36.8%) non-survivors. Clinical details of horses enrolled in the study, stratified by type of colic, including diagnosis, treatment, and outcome are reported in Table S1.

Data concerning the SIRS status and SIRS score (0-6 point-scale) divided based on type of colic are reported in Table 2. Data about BIOs concentrations from healthy horses, horses affected by NSC and SC are summarized in Table 3. Descriptive statistics for all oxidative and antioxidant biomarkers, stratified by colic type and survival outcome, are presented in Table S2.

**Table 2:** Systemic inflammatory response syndrome (SIRS) status and corresponding 0-6 point-scale SIRS scores in horses with non-strangulating colic (NSC) and strangulating colic (SC). The table displays the distribution of SIRS-positive and SIRS-negative cases within each colic type and the number of horses at each point score level.

Type of colic	SIRS status	0-6 point-scale SIRS score
**NSC (*n* = 42)**	15/42 (35.7%) SIRS-negative	0/6 8/15 (53.3%)
		1/6 7/15 (46.7%)
	27/42 (64.3%) SIRS-positive	2/6 6/27 (22.2%)
		3/6 9/27 (33.3%)
		4/6 8/27 (29.7%)
		5/6 4/27 (14.8%)
**SC (*n* = 19)**	3/19 (15.8%) SIRS-negative	0/6 2/3 (66.7%)
		1/6 1/3 (33.3%)
	16/19 (84.2%) SIRS-positive	2/6 4/16 (25%)
		3/6 6/16 (37.5%)
		4/6 4/16 (25%)
		5/6 2/16 (12.5%)

**Table 3:** Comparison of oxidative stress and inflammation-related biomarkers among healthy horses (H), horses with non-strangulating colic (NSC), and horses with strangulating colic (SC). Data are presented as medians with IQRs. Pairwise *P*-values indicate statistical significance between groups (H vs NSC/H vs SC/NSC vs SC).

Variable (unit)	H (*n* = 10)	NSC (*n* = 42)	SC (*n* = 19)	Pairwise *P*-values (H vs NSC)	Pairwise *P*-values (H vs SC)	Pairwise *P*-values (NSC vs SC)
**Lactate (mmol/L)**	1.00 (0.80-1.05)	2.20 (1.60-3.30)	1.90 (1.80-2.70)	<.001	<.001	1
**AREase (kU/L)**	296.11 (268.06-371.39)	477.78 (348.61-605.84)	481.67 (398.33-609.44)	.01	.01	1
**POase (kU/L)**	81.09 (72.20-102.06)	48.10 (37.05-56.19)	43.55 (36.40-59.15)	<.001	<.001	1
**LPO (MDA/μL)**	7.07 (5.95-10.01)	6.98 (5.35-8.93)	7.13 (6.19-8.52)	1	1	1
**SOD (U/mL)**	2.71 (2.41-3.21)	2.83 (2.41-3.24)	2.35 (2.08-2.59)	1	.28	.04
**BChE (μmol/mL/min)**	13.59 (11.99-15.56)	11.62 (9.63-15.49)	13.06 (8.56-17.37)	1	1	1
**TAC (μmol/mL)**	3.75 (2.38-5.13)	8.00 (2.75-10.50)	8.00 (5.50-10.00)	.06	.02	.95
**GST (U/mg protein)**	8.62 (6.97-9.56)	19.45 (11.55-30.26)	20.92 (13.48-27.20)	.001	.001	1
**GPx (U/mg protein)**	0.16 (0.09-0.23)	0.52 (0.29-0.70)	0.57 (0.42-0.85)	.001	.001	1

The GLMM identified significant fixed effects for several BIOs across different predictors. For AREase, activity differed between survivors and non-survivors, with higher AREase activity observed in survivors (coefficient [coef.] 106.65 kU/L; 95%CI, 24.70-188.60; *P* = .01). No significant differences were detected across time points or colic type. For LPO, activities differed by colic type, with lower LPO activity in horses with NSC compared with those with SC (coef. −1.24 MDA/μL; 95%CI, −2.81 to −0.32; *P* = .01). No significant differences were observed across time points, or survival groups. For SOD, activity differed by both colic type and survival group. Horses with NSC had higher SOD activity compared with those with SC (coef. 0.42; 95%CI, 0.03-0.81; *P* = .04), whereas survivors had lower SOD activity than non-survivors (coef. −0.38 U/mL; 95%CI, −0.76 to −0.06; *P* = .03). No significant differences were observed across time points. For TAC, activities also differed by colic type and survival group. Horses with NSC had higher TAC activity compared with those with SC (coef. 1.21; 95%CI, 0.10-2.98; *P* = .04), and survivors exhibited lower TAC activity compared with non-survivors (coef. −3.37 μmol/mL; 95%CI, −5.49 to −1.25; *P* = .01). A difference was also observed at T0 (coef. 2.57 μmol/mL; 95%CI, 3.67-9.32; *P* = .04), whereas no other time-related effects were significant. For POase, BChE, GST, and GPx, no significant differences were found across time points, colic type, or survival groups.

## Discussion

We aimed to evaluate BIOs involved in oxidative stress in healthy horses and those with NSC or SC, and to assess the relationship between BIOs activity and clinical outcomes.

Overall, BIOs analysis identified significant differences among healthy, NSC, and SC groups for blood lactate, AREase, POase, SOD, TAC, GST, and GPx. The GLMM further identified significant associations among colic type, survival status, and sampling time points for specific BIOs. Blood lactate concentrations were significantly increased in horses affected by both NSC and SC compared with healthy horses, but no significant differences were observed between NSC and SC groups, or between survivors and non-survivors. These findings agree with previous reports showing higher lactate in colic horses compared with healthy ones,^21^^,^^32^ but contrast with studies reporting higher concentrations in SC and in horses with poor prognosis.^33–37^ Increase in lactate concentration is primarily related to bowel ischemia but also can be influenced by additional factors such as dehydration, hypovolemia, and endotoxemia, which may occur in both colic types.^33^ We did not specifically evaluate the effects of dehydration, hypovolemia, or endotoxemia. Therefore, it is not possible to determine whether these conditions contributed to the lactate concentrations observed in the enrolled population. In addition, the rate at which lactate diffuses from a necrotic intestinal segment into peritoneal fluid and then into systemic circulation is not well established.^33^ The relatively low number of strangulating colic cases may have limited our ability to detect differences. Similarly, the lack of differences between survivors and non-survivors may be a consequence of the small number of non-survivors in the cohort.

Activity of AREase was significantly lower in healthy horses compared with those affected by NSC and SC. In human medicine, studies on AREase activity in gastrointestinal disorders have yielded conflicting results, with reports of unchanged, decreased, or even increased activity depending on the condition and inflammatory status.^38^^,^^39^ In animals, serum AREase activity has been rarely investigated, with some studies reporting decreases in camels with mite infestation and in cows with mastitis or parasitic infections.^40^^,^^41^

To date, no studies have evaluated AREase activity in horses, and the observed discrepancy between our findings and those reported in the literature may be attributed to several factors, including species-specific variations, differences in the pathological conditions examined, and variations in the methodologies employed to evaluate AREase activity. Horses that survived had higher AREase activity compared with those that did not survive, with the GLMM identifying a significant effect of survival status on AREase activity. In humans, higher serum AREase activity has been related to a favorable outcome in patients affected by ischemic stroke,^42^ and several studies have reported that AREase activity protects low-density lipoproteins and high-density lipoproteins from oxidation.^43–45^ Therefore, AREase also may have a similar protective effect in horses.

In our study, POase activity was significantly lower in horses with NSC and SC compared with healthy horses. The POase activity in horses has been further explored in recent years, leading to the recognition of preanalytical variability and the validation of reliable assay systems for the horses.^46^^,^^47^ Decreased serum PON-1 activity has been observed in horses with *Theileria equi* infection,^6^ experimental induced endotoxemia,^7^ and inflammation of different causes and variable severity.^8^ Moreover, a recent study identified a significant difference in POase activity between horses with colitis and healthy controls.^48^

Colic type significantly influenced LPO activity, with NSC linked to lower concentration than SC. This finding is consistent with previous studies of horses reporting higher LPO activity in horses with flatulent and impaction colic compared with spasmodic colic and healthy controls,^10^ and increased LPO in strangulating colic compared with non-strangulating colic.^49^ These findings support the role of oxidative stress in colic pathophysiology, with more severe or ischemic conditions such as strangulating colic associated with more lipid peroxidation.

A significant decrease of SOD activity was found in horses afflicted by SC compared with those affected by NSC. Although no previous studies have directly assessed SOD activity in relation to colic type, decreases in SOD have been reported in several pathological conditions of horses. For instance, in horses affected by equine infectious anemia, SOD activity was found to decrease progressively over time after seroconversion, with long-term infected animals showing notably lower SOD activity.^50^ Lower SOD activity has been documented in horses with peritonitis,^11^  *Clostridium difficile*-induced enterocolitis,^12^ colitis secondary to phenylbutazone administration,^13^ and EGUS^14^ compared with healthy controls. Such decreases have been linked to the accumulation of oxidative damage products induced by reactive oxygen and nitrogen species.^49^ Therefore, the lower SOD activity observed in the SC group may reflect a more severe oxidative stress status associated with this condition. Moreover, in our study, surviving horses had lower SOD activity compared with those that did not survive. This finding contrasts with the literature, where SOD activity has been reported to significantly decrease in non-surviving colic-affected horses compared with survivors.^11^^,^^14^ The discrepancy might be associated with several factors such as different timing of sampling, different treatments, differences in colic severity or laboratory assay differences.

In our study, TAC concentration was significantly higher in horses with NSC and SC compared with healthy ones. In previous studies, lower TAC activity has been reported in horses during enterocolitis,^12^ EGUS,^14^ leptospirosis,^51^ metritis,^52^ and rhabdomyolysis.^53^ Although lower TAC has been reported in other disorders of horses, the interpretation of TAC depends strongly on the oxidative context. Indeed, TAC alone does not directly reflect oxidative stress level, because increased TAC may occur as part of an adaptive antioxidant response to increased pro-oxidant production.^54^ Increased TAC has been reported as a possible compensatory mechanism triggered by oxidative stress.^55^ The higher TAC results observed in colic-affected horses might be consistent with a reactive upregulation of circulating antioxidants in response to oxidative stress stimuli.

In addition, a significant time-related difference was observed, with TAC results at T0 being higher than at later sampling points. Increases in plasma total antioxidant status have been observed in horses subjected to 8-h road transport. This transient increase is thought to result from the redistribution of small molecule antioxidants in response to oxidative stress.^56^ In the colic population enrolled in our study, the transient increase at admission similarly might reflect acute mobilization of antioxidant defenses at the onset of disease, followed by partial consumption or redistribution as oxidative stress progresses or as treatment begins.

Both GST and GPx activities were significantly lower in healthy horses compared with those afflicted by NSC and SC. In humans, GST has proven useful in assessing intestinal ischemia. Specifically, GST activity has been reported to be increased in patients with intestinal ischemia compared with healthy controls.^15–17^ In veterinary medicine, GST has been poorly studied. In horses, the only study conducted in adult horses found no significant changes in GST activity after prolonged transportation.^18^ In our study, the result appears to be more in line with studies on humans than with those in horses. The discrepancy with the transportation study may reflect that, although prolonged transportation is a recognized stressor, it may not elicit the same oxidative changes as do pathological conditions such as colic. In addition, GST expression might be upregulated during oxidative stress as part of the phase II detoxification response, catalyzing the conjugation of glutathione with reactive electrophilic metabolites of cellular damage.^57^ The increased GST activity observed in colic-affected horses thus may represent both enzyme release from injured tissues and adaptive induction of antioxidant defenses in response to heightened oxidative challenge.

Research on GPx activity in adult horses during long-distance transport and strenuous physical activity has produced inconsistent findings. Although some studies reported increased GPx activity in horses undergoing strenuous effort,^19^^,^^20^ others found a decrease in activity.^18^ In our study, GPx activity was significantly lower in healthy horses compared with those afflicted by NSC and SC. This pattern is consistent with evidence that, similar to GST, GPx expression is upregulated during oxidative stress as part of an adaptive enzymatic defense mechanism.^58^ The increased GPx activity observed in colic-affected horses therefore might reflect a compensatory antioxidant response to oxidative stress rather than inherently higher antioxidant status.

Although several BIOs showed significant differences between healthy and colic-affected horses, other comparisons did not reach statistical significance. AREase did not differ by colic type or time point; LPO showed no differences across time or survival groups; SOD exhibited no time-related changes; TAC varied only at T0; and POase, BChE, GST, and GPx did not differ across time points, colic type, or survival groups. These findings might reflect the complex and time-dependent kinetics of oxidative and antioxidant responses in horses. Antioxidant enzymes such as GPx and GST may require transcriptional activation and could have delayed or transient responses that our sampling schedule may not have captured.^57^^,^^58^ Considerable interindividual variability in enzyme activity also has been reported in horses,^59^ which could further attenuate differences among clinical categories. Finally, pharmacological treatment before admission may have introduced additional variability.

A limitation of our study is the smaller number of horses constituting the “type of colic” and “outcome” subgroups. In addition, the number of sampled horses decreased over time because of discharges, death, or euthanasia occurring before 96 h post-admission. Moreover, the sample size was not determined a priori, because no previous data were available to estimate the expected variability and effect sizes for several of the investigated BIOs. Although post hoc analyses indicated that the study was sufficiently powered to detect large effects, BIOs with small or moderate effect sizes likely were underpowered. This limitation reinforces the exploratory and hypothesis-generating nature of the study and highlights the need for future research with larger, prospectively designed cohorts. The horses in our study were admitted at VTHs after spontaneous onset of colic symptoms but, because of the clinical nature of the study, it was not possible to pinpoint the exact timing of the onset of SIRS, making it challenging to standardize the sampling schedule. Moreover, some horses underwent laparotomy as part of the treatment for colic. Laparotomy itself can induce an inflammatory response^60^ and may therefore have influenced the activity and concentrations of some BIOs. Because horses from both the NSC and SC groups underwent surgery and, surgical interventions were carried out within an analogous timeframe, this potential bias may have been partially mitigated. However, the effect of laparotomy on BIO activities was not specifically evaluated. Future studies should investigate the influence of surgery on these BIOs to better distinguish changes related to colic from those associated with the surgical intervention.

The inclusion of healthy mares from a reproductive herd as controls represents a potential source of selection bias, because these animals may not be fully representative of the broader clinical population from which colic cases were drawn. In particular, the control group consisted exclusively of females, whereas the colic population included horses of both sexes. Therefore, we cannot exclude a contribution of sex-related differences in oxidative and antioxidant BIOs to some of the observed contrasts between healthy and colic-affected horses. However, our primary aim was descriptive, namely, to characterize oxidative and antioxidant status across different clinical conditions rather than to establish diagnostic cut-offs or prognostic thresholds. Controls therefore were used to provide baseline reference values for comparison, rather than as a strictly matched population. In addition, some colic cases originated from the same reproductive herd, which may partially minimize population differences, although this factor does not fully address the potential impact of sex imbalance. These limitations should be considered when interpreting comparisons between healthy and colic-affected horses. A fully sex-stratified analysis was not pursued because subdivision of the sample by sex, colic type, and outcome would have resulted in very small subgroups, precluding meaningful statistical inference. Future studies specifically designed to evaluate sex effects, with balanced recruitment of males and females in both healthy and colic populations, are warranted. In addition, multiple BIOs were evaluated across several clinical categories, and no correction for multiple testing was applied. This decision reflects the exploratory scope of the study and the biological independence of the BIOs assessed. Therefore, significant associations should be interpreted cautiously and considered preliminary, serving primarily to generate hypotheses for future confirmatory research.

### Conclusion

We identified significant associations between oxidative stress BIOs and colic in horses, with distinct variations in BIOs concentrations observed between healthy horses and those affected by NSC or SC. The findings suggest that differences in BIOs activity may be linked to processes such as ischemic damage, inflammation, and antioxidant response mechanisms. The results may indicate a relationship between BIOs concentration and clinical outcomes, but this link needs to be further investigated by increasing the number of horses in both survivor and non-survivor groups. Although the findings align with previous literature in many aspects, certain discrepancies suggest the need for further research. Future studies with larger cohorts and standardized methodologies are needed to confirm these findings and refine the clinical application of BIOs as diagnostic and prognostic tools in horses with colic.

## Supplementary Material

REV_4_Supplementary_tables_aalag017

## Off-label antimicrobial declaration

The authors declare no off-label use of antimicrobials.

## Institutional animal care and use committee or other approval declaration

Approved by the Ethical Committee of the University of Pisa (Prot. N. 03/2023) and owners written consent for the plasma collection obtained for all the horses included in this study.

## Abbreviations

AIC: Akaike’s information criterion
AREase: arylesterase
BIOs: biomarkers
BChE: butyrylcholinesterase
BSA: bovine serum albumin
BTChE: butyrylthiocholinesterase
CDNB: 1-chloro-2,4-dinitrobenzene
Coef: coefficient
EGUS: equine gastric ulcer syndrome
GLMM: generalized linear mixed model
GPx: glutathione peroxidase
GSH: reduced glutathione
GST: glutathione S-transferase
HEPES: 4-(2-hydroxyethyl)-1-piperazineethanesulfonic acid
LPO: lipid peroxidation
MDA: malondialdehyde
NSC: non-strangulating colic
PON1: paraoxonase 1
POase: paraoxonase
SC: strangulating colic
SIRS: systemic inflammatory response syndrome
SOD: superoxide dismutase
TAC: total antioxidant capacity
TBA: thiobarbituric acid
TCA: trichloroacetic acid
TPTZ: 2,3,5-triphenyl-1,3,4-triaza-2-azoniacyclopenta-1,4-diene chloride
VTHs: veterinary teaching hospitals
