# Supplementary material for: Evaluation of oxidative stress and antioxidant defense biomarkers in healthy and colic horses: correlation with type of colic and outcome
Source: J Vet Intern Med. 2026 Feb 14;40(1):aalag017. doi: 10.1093/jvimsj/aalag017 (PMC12906274; doi:10.1093/jvimsj/aalag017)
Supplement: REV_4_Supplementary_tables_aalag017 [file rev_4_supplementary_tables_aalag017.docx]

***Supplementary Table 1.*** Details of horses enrolled in the study, stratified by type of colic (non-strangulating colic, NSC; strangulating colic, SC), including diagnosis, treatment (medical, M; surgical, S), and outcome (discharged, D; euthanized, E).

| **Patient** | **Type of colic** | **Diagnosis** | **Treatment** | **Outcome** |
| --- | --- | --- | --- | --- |
| 1 | NSC | Small intestine impaction | S | D |
| 2 | NSC | Small intestine and pelvic flexure impaction | S | E |
| 3 | NSC | Small intestine impaction | S | D |
| 4 | NSC | Small intestine impaction | S | D |
| 5 | NSC | Small intestine impaction | S | D |
| 6 | NSC | Small intestine impaction | S | D |
| 7 | NSC | Small intestine impaction | S | D |
| 8 | NSC | Small intestine impaction | S | D |
| 9 | NSC | Large colon impaction (sand) | S | D |
| 10 | NSC | Small intestine impaction | S | D |
| 11 | NSC | Small intestine impaction | S | D |
| 12 | NSC | Pelvic flexure impaction | S | D |
| 13 | NSC | Small intestine and pelvic flexure impaction | S | D |
| 14 | NSC | Large colon impaction (ascarid) | S | D |
| 15 | NSC | Small intestine and pelvic flexure impaction | S | D |
| 16 | NSC | Large colon impaction (sand) | M | D |
| 17 | NSC | Small intestine impaction | M | D |
| 18 | NSC | Pelvic flexure impaction | M | D |
| 19 | NSC | Small colon impaction | M | D |
| 20 | NSC | Pelvic flexure impaction | M | D |
| 21 | NSC | Small colon impaction | M | D |
| 22 | NSC | Pelvic flexure impaction | M | E |
| 23 | NSC | Pelvic flexure impaction | M | D |
| 24 | NSC | Pelvic flexure impaction | M | D |
| 25 | NSC | Pelvic flexure impaction | M | D |
| 26 | NSC | Pelvic flexure impaction | M | D |
| 27 | NSC | Pelvic flexure impaction | M | D |
| 28 | NSC | Large colon impaction (sand) | M | D |
| 29 | NSC | Pelvic flexure impaction | M | D |
| 30 | NSC | Pelvic flexure impaction | M | D |
| 31 | NSC | Small intestine impaction | M | D |
| 32 | NSC | Pelvic flexure impaction | M | D |
| 33 | NSC | Pelvic flexure and small colon impaction | M | D |
| 34 | NSC | Small intestine and pelvic flexure impaction | M | D |
| 35 | NSC | Pelvic flexure impaction | M | D |
| 36 | NSC | Pelvic flexure impaction | M | D |
| 37 | NSC | Pelvic flexure impaction | M | E |
| 38 | NSC | Pelvic flexure impaction | M | D |
| 39 | NSC | Pelvic flexure impaction | M | D |
| 40 | NSC | Pelvic flexure impaction | M | D |
| 41 | NSC | Pelvic flexure and small colon impaction | M | D |
| 42 | NSC | Pelvic flexure impaction | M | D |
| 43 | SC | Large colon dislocation | S | D |
| 44 | SC | Pedunculated lipoma | S | E |
| 45 | SC | Nephrosplenic entrapment of the colon | S | D |
| 46 | SC | Small intestine volvulus | S | E |
| 47 | SC | Post-celiotomy adhesions | S | E |
| 48 | SC | Large colon dislocation | S | D |
| 49 | SC | Large colon dislocation | S | D |
| 50 | SC | Nephrosplenic entrapment of the colon | S | D |
| 51 | SC | Small intestine volvulus | S | D |
| 52 | SC | Nephrosplenic entrapment of the colon | S | D |
| 53 | SC | Large colon volvulus | S | E |
| 54 | SC | Large colon volvulus | S | E |
| 55 | SC | Small intestine volvulus | S | D |
| 56 | SC | Large colon volvulus | S | D |
| 57 | SC | Large colon volvulus | S | E |
| 58 | SC | Small intestine volvulus | S | D |
| 59 | SC | Large colon volvulus | M | E |
| 60 | SC | Large colon dislocation | M | D |
| 61 | SC | Nephrosplenic entrapment of the colon | M | D |

***Supplementary Table 2.*** Median (25^th^ and 75^th^ percentile) values of oxidative and antioxidant biomarkers measured in horses with colic, grouped according to survival outcome (survivors and non-survivors) and colic type (non-strangulating colic, NSC; strangulating colic, SC). Units of measurement are indicated for each biomarker.

| **Biomarker (unit)** | **NSC** | **SC** | **Survivors** | **Non-survivors** |
| --- | --- | --- | --- | --- |
| AREase (kU/L) | 472.2 (343.6–596.9) | 398.3 (300.6–606.7) | 471.7 (348.3–615.0) | 341.1 (253.6–512.5) |
| POase (kU/L) | 47.6 (38.0–56.1) | 41.6 (37.1–51.7) | 46.1 (37.6–55.8) | 41.6 (37.9–59.2) |
| LPO (MDA/μL) | 7.5 (5.8–9.7) | 8.3 (6.8–10.0) | 7.7 (6.1–9.7) | 7.9 (6.3–9.7) |
| SOD (U/mL) | 2.64 (2.32–3.13) | 2.41 (2.21–3.01) | 2.56 (2.28–3.09) | 2.85 (2.26–4.11) |
| BChE (µmol/mL/min) | 10.72 (7.25–13.13) | 10.93 (6.35–14.92) | 10.93 (6.96–14.33) | 10.49 (7.25–11.96) |
| TAC (µmol/mL) | 6.0 (4.0–9.3) | 6.0 (4.5–9.0) | 5.5 (4.0–8.5) | 9.0 (4.5–11.0) |
| GST (U/mg protein) | 14.01 (9.39–23.53) | 15.79 (12.21–23.89) | 14.21 (9.58–22.39) | 16.61 (12.41–38.26) |
| GPx (U/mg protein) | 0.53 (0.26–0.81) | 0.61 (0.40–0.90) | 0.56 (0.33–0.84) | 0.61 (0.37–0.88) |
